# Early initiation of breastfeeding is inversely associated with public and private c-sections in 73 lower- and middle-income countries

**DOI:** 10.1038/s41598-022-25564-w

**Published:** 2022-12-06

**Authors:** Juliana S. Vaz, Giovanna Gatica-Domínguez, Paulo A. R. Neves, Luís Paulo Vidaletti, Aluísio J. D. Barros

**Affiliations:** 1 International Center for Equity in Health, Federal University of Pelotas, Rua Marechal Deodoro, 1160, 3rd Floor, Pelotas, RS 96020-220 Brazil; 2 Faculty of Nutrition, Federal University of Pelotas, Pelotas, Brazil

**Keywords:** Epidemiology, Nutrition

## Abstract

Although studies in low- and middle-income countries (LMICs) have examined the effects of c-sections on early initiation of breastfeeding (EIBF), the role of the place of birth has not yet been investigated. Therefore, we tested the association between EIBF and the type of delivery by place of birth. Data from 73 nationally representative surveys carried out in LMICs between 2010 and 2019 comprised 408,013 women aged 15 to 49 years. Type of delivery by place of birth was coded in four categories: home vaginal delivery, institutional vaginal delivery, c-section in public, and c-section in private health facilities. We calculated the weighted mean prevalence of place of birth and EIBF by World Bank country income groups. Adjusted Poisson regression (PR) was fitted taking institutional vaginal delivery as a reference. The overall prevalence of EIBF was significantly lower among c-section deliveries in public (PR = 38%; 95% CI 0.618–0.628) and private facilities (PR = 45%; 95% CI 0.54–0.566) compared to institutional vaginal deliveries. EIBF in c-sections in public facilities was slightly higher in lower-middle (PR = 0.650, 95% CI 0.635–0.665) compared to low (PR = 0.544, 95% CI 0.521–0.567) and upper-middle income countries (PR = 0.612, 95% CI 0.599–0.626)**.** EIBF was inversely associated with c-section deliveries compared to institutional vaginal deliveries, especially in private facilities compared to public ones.

## Introduction

Early initiation of breastfeeding (EIBF)—putting a newborn to the breast within the first hour after birth—ensures that the infant receives essential protection factors via colostrum, which reduces the risk of infections and neonatal mortality. EIBF triggers the release of essential hormones, such as oxytocin, which promotes uterine contraction and reduces the risk of postpartum hemorrhage, and prolactin, which is necessary for milk production^1^. It also increases the likelihood of exclusive breastfeeding and the overall duration of breastfeeding^2,3^, with both having long term beneficial effects on child development and protection against non-communicable diseases in adulthood^2–4^.

According to UNICEF, EIBF increased from 32 to 46% between 2000 and 2015 in low- and middle-income countries (LMICs), which means that nearly 77 million children (out of 140 million) were not breastfed within the first hour after birth in these countries. With respect to regions of the world, greater progress was seen in South Asia (from 16 to 45%), while in West & Central Africa, modest achievements were documented (from 33 to 35%)^5^.

Amidst several factors that influence EIBF, cesarean section deliveries (hereinafter, c-sections) have been systematically and directly associated with delayed initiation of breastfeeding^6–8^. Some obsolete practices in health facilities and the lack of knowledge about EIBF after c-sections inhibit that children are put to breast after birth^2^. Furthermore, it likely eases those children to receive prelacteal feedings that have detrimental influence on later breastfeeding practices^2,9^. This is of great concern given the unprecedented increase in the number of c-sections globally (from 12.1% in 2000 to 21.1% in 2015), especially in some regions of the world, such as South Asia, Eastern Europe & Central Asia, East Asia & the Pacific, Latin America & Caribbean, and Western Europe^10^.

To protect, promote and support optimal breastfeeding practices, WHO and UNICEF launched the Baby Friendly Hospital Initiative to motivate health facilities worldwide to follow the *Ten Steps to Successful Breastfeeding*. Step 4 encourages early skin-to-skin contact between the mother and newborn and supports EIBF. Both organizations also recognize the importance of including the private sector health facilities in the initiative^11^. It is noteworthy that such health units represent the highest share of c-sections among institutional deliveries, being 1.6 times more frequent than in deliveries from public facilities, shedding light on the importance of such units in the increase of EIBF globally^10^. C-sections may negatively affect the success of the Baby-Friendly Hospital Initiative by reducing breastfeeding practices, regardless of whether it is a public or private institution^12,13^.

Although several studies in LMICs have examined the relationship between type of delivery and timing of breastfeeding initiation, most have focused on the detrimental effects of c-sections on EIBF; however, the role of the place of birth on the EIBF has not yet been widely investigated in multi-country analyses of LMICs. As such, we sought to investigate the association between EIBF and the type of delivery by place of birth covering a large number of national health surveys in LMICs.

## Methods

### Data sources

This is a multi-country, cross-sectional survey analysis study. We used data of publicity available microdata from large scale, nationally representative cross-sectional surveys carried out in LMICs, namely Demographic Health Surveys (DHS)^14^ and Multiple Indicator Cluster Surveys (MICS)^15^ since 2010 or later. These surveys are highly comparable in terms of sampling methods, questionnaires, measurements, and field procedures^16^. Both surveys employ multistage sampling strategies to collect data at the household level, through face-to-face interviews with women of childbearing age (15–49 years) using standardized questionnaires administered by trained field workers. The similarity in study design and implementation quality between DHS and MICS allows direct comparisons between surveys and between countries. We also included the nationally representative *Encuesta Nacional de Salud y Nutrición* (National Health and Nutrition Survey—NSS) conducted in Ecuador in 2012, after harmonizing its data set and variables in accordance with the DHS/MICS default^17^.

Data for 104 surveys were available in the International Center for Equity in Health (ICEH) database (http://www.equidade.org) at the time of analysis. We restricted the investigation to countries with complete data to adequately generate the exposure variable and to those with all covariables to adjust the models. Additionally, we excluded countries with an insufficient number of observations on private c-sections or in-home delivery which models did not convert properly (Supplementary Fig. 1). Thus, the present analysis included 73 LMICs.

Countries were grouped according to the World Bank’s income classification in the year of survey implementation^18^. Supplementary Table 1 details the countries included in the analysis, their income, and the sample size of women. The design of the survey group sample was considered when performing the analyzes using the ‘svy’ command in Stata. Analyzes were weighted by the national population size of women of reproductive age (15–49 years) in the year the surveys were carried out using the World Bank Population Estimates and Projections^18^.

### Exposure

We used the following questions used in DHS/MICS questionnaires to determine the exposure investigated: i) ‘Where did you give birth to (NAME)?’, which classifies the place of delivery as ‘at home’, considering the woman’s place of residence or any other house, or in a ‘health facility’, including health centers or clinics of government and private hospitals, and ii) ‘Was (NAME) delivered by cesarean, that is, did they cut your belly open to the baby out?’ and provides a dichotomous answer (yes, no). Both questions are self-reported, and data have been shown to be valid and reliable^19,20^. Then, we were able to code the type of delivery by place of birth into four categories: (i) home vaginal delivery, (ii) institutional vaginal delivery, (iii) c-section in public facility, and (iv) c-section in private facility. All live births in the two years prior to the survey were deemed in the analysis. In case of women with multiple births during the reference period, only the most recent birth was considered.

### Outcome

The prevalence of early initiation of breastfeeding (EIBF) was calculated based on the WHO definition^21,22^ and estimates to the proportion of children born in the last 24 months who were put to the mother’s breast within 1 h after birth. EIBF was categorized as a dichotomous variable; ‘Yes’, child who initiated breastfeeding within 1 h of birth and ‘No’, child who initiated breastfeeding after that.

### Potential confounders

Place of residence (urban or rural) was determined using country-specific criteria adopted by national governments at the time of the survey^14,15^. Maternal age refers to the completed age of the woman at the time of the survey. Women’s formal education attainment was classified into three categories: none (no formal education); primary (7 years or less); secondary or higher (8 years or more), according to country-specific information provided in the datasets. A wealth index was generated through principal component analysis, considering the presence of household assets, building materials, and utilities like water and electricity, and adjusted for place of residence^23^. The final index was split into quintiles, where the first quintile represents the 20% poorest and the fifth quintile the 20% richest.

### Statistical analysis

We performed a country-level analysis to calculate the weighted prevalence (95% CI) of the place of delivery and EIBF, later summarized by country income groups and place of delivery. Using individual-level data, we calculated the crude and adjusted prevalence ratios using Poisson regression models. Additionally, we calculated prevalence ratios comparing EIBF and type of delivery by place of birth, considering institutional vaginal delivery as benchmark. Models were adjusted for place of residence, maternal age, maternal formal education, and household wealth. Meta-analyses were performed to summarize the crude and adjusted effects of type of delivery by place of birth on EIBF, as well as by country income groups. The heterogeneity test (I^2^) was applied to assess the variability in the estimates between countries, and the magnitude of heterogeneity was classified using the following criteria: homogeneous (≥ 25%), moderately heterogeneous (26–74%), and highly heterogeneous (≥ 75%)^24^. To assess whether the type of survey (DHS, MICS or NSS) had an impact on the findings, a meta-regression was carried out including this variable to estimate the adjusted R^2^. This parameter provides the proportion of variance between studies explained by a covariate. If the adjusted R^2^ is negative, the covariates explain less of the heterogeneity than would be expected by chance^25^.

All analyzes were performed in Stata 16.0 (Stata Corp, College Station, TX, USA). Ethical clearance was responsibility of the national institutes in charge of data collection each country.

## Results

The earliest surveys were carried out in Burkina Faso, Central African Republic, and Colombia in 2010, while the latest were in Bangladesh, Zimbabwe, and Thailand in 2019, with a median year of 2016. The analytical sample consisted of 408,013 women aged 15–49 years, with survey sample sizes ranging from 916 women (Belize) to 101,910 women (India) (Supplementary Table 1). The studied countries represented 93% of low, 51% of lower middle, and 36% of upper middle-income countries.

### Birth deliveries according to place of birth

In the pooled analysis, institutional and home vaginal deliveries represented 55.2% and 24.5% of cases, while c-sections in private and public facilities represented 10.9% and 9.4%, respectively. On average, c-section prevalence in public facilities accounted for 25.7%, 6.6%, and 3.7% in upper middle, lower middle, and low-income countries, respectively. C-sections prevalence in private facilities accounted for 10.4% and 12.9% in upper and middle-income countries, respectively, but represented 1.2% in countries with low income (Table 1). Supplementary Table S1 presents the national weighted prevalence of type of delivery by place of birth according to the income country group and shows a wide range in the c-section rates across countries. A contrasting practice of c-sections between public and private facilities was observed within countries. In lower-middle income countries, El Salvador (29.9%) and Mongolia (25%) had one of the highest prevalence in c-section in public facilities, but surprisingly very low prevalence in private facilities, respectively 1.6% and 1.1%. In upper middle-income countries, the prevalence in c-sections in public and private facilities contracted widely in Colombia (37.6% vs. 0.1%) and Albania (29.2% vs. 2.5%), while high prevalence of c-sections in both sectors were observed in the other countries, such as the Dominican Republic (32.9% vs. 25,3%), Turkey (22.2% vs. 24.6%), and Ecuador (25.2% vs. 15.6%), respectively, in public and private.

**Table 1:** Weighed^a^ mean prevalence of place of birth and early initiation of breastfeeding for 73 countries with available household surveys^b^ from 2010–2019.

	All countries		Low income		Lower-middle income		Upper-middle income	
	%	(95% CI)	%	(95% CI)	%	(95% CI)	%	(95% CI)
**Place of birth**								
Home vaginal delivery	24.5	(23.0; 25.9)	38.3	(35.5; 41.2)	25.4	(24.2; 26.7)	6.3	(5.4; 7.5)
Institutional vaginal delivery	55.2	(53.5; 57.0)	56.7	(53.9; 59.5)	54.5	(53.2; 55.8)	57.6	(54.5; 60.6)
C-section public facilities	9.4	(8.5; 10.4)	3.7	(3.0; 4.6)	7.2	(6.6; 7.9)	25.7	(23.2; 28.2)
C-section private facilities	10.9	(10.1; 11.9)	1.2	(0.9; 1.8)	12.9	(12.1; 13.7)	10.4	(8.7; 12.5)
**Early initiation of breastfeeding**								
Home vaginal delivery	45.8	(40.8; 50.4)	53.7	(49.8; 57.6)	42.4	(39.9; 45.0)	55.2	(37.0; 70.5)
Institutional vaginal delivery	52.5	(50.3; 54.7)	62.1	(59.1; 65.0)	48.7	(46.9; 50.5)	62.1	(58.3; 65.7)
C-section public facilities	31.8	(27.3; 37.0)	27.1	(18.9; 37.7)	31.5	(27.8; 35.7)	37.9	(32.9; 43.2)
C-section private facilities	28.2	(21.6; 38.1)	29.0	(14.6; 50.9)	27.4	(23.2; 33.2)	31.4	(20.1; 50.5)
N of countries	73		25		28		20	

^a^Weighed for national population of women of reproductive age (15–49 years); source: World Bank, 2020.

^b^Multiple Indicator Cluster Survey, Demographic Health Survey and National Health and Nutrition Survey (Ecuador).

*C-section* cesarean section; *CI* confidence interval.

### EIBF according to place of delivery and income groups

EIBF prevalence varied substantially according to the place of birth and income groups (Table 1). In the pooled analysis, EIBF was higher in institutional vaginal deliveries (52.5%) and home deliveries (45.8%), and lower in c-sections in public (31.8%) and private (28.2%) facilities. According to income groups, lower-middle income countries had the lowest EIBF prevalence in institutional vaginal deliveries (48.7%) and home vaginal deliveries (42.4%). EIBF was significantly lower in c-section deliveries in all income groups, ranging from 27.1% to 37.9% in public facilities to 27.4% to 31.4% in private facilities. EIBF prevalence varied substantially between and within countries, showing that an expressive number of children did not receive breastmilk in the first hour after birth (Supplementary table S2). In most LMICs, the EIBF prevalence was below 25% in c-sections in public and private facilities. In upper-middle income countries, five out of 20 countries (Belize, Maldives, Namibia, Turkey, and South Africa) had EIBF prevalence in c-sections greater than 50% in both public and private facilities.

### Prevalence ratio of EIBF according to income groups stratified by place of birth

**Figure 1:**
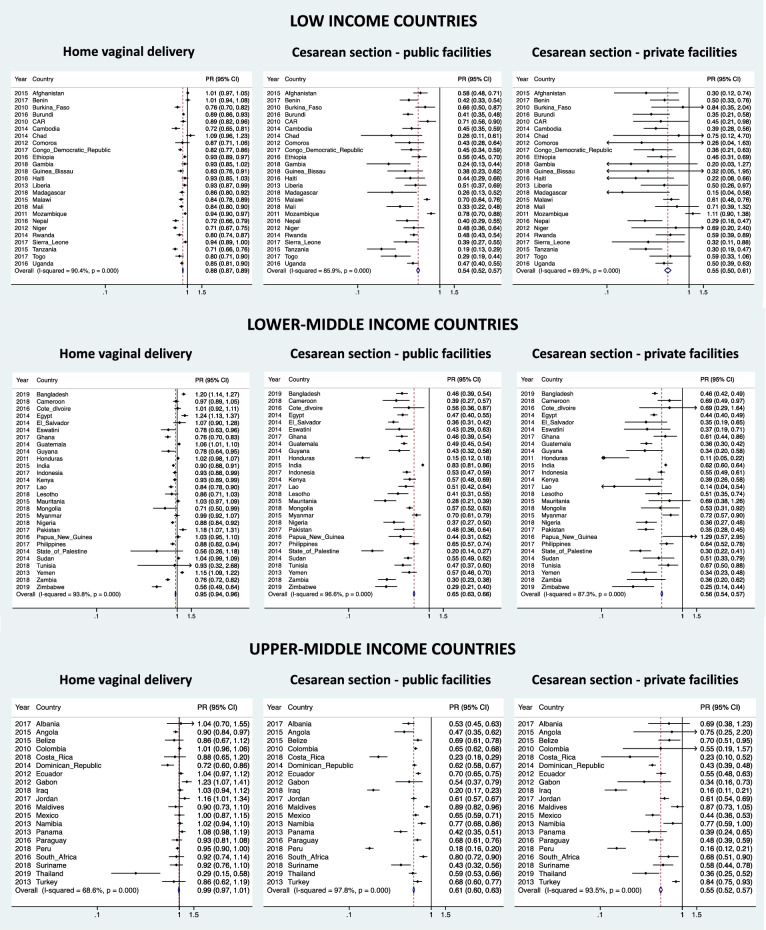
Prevalence ratio (PR) of early breastfeeding by type of delivery and place of birth of 73 countries by income country, taking institutional vaginal delivery as reference. *Note* Adjusted for living area, maternal education, maternal age, and wealth quintiles.

Adjusted pooled prevalence ratio coefficients showed that c-section deliveries in public and private facilities had a significantly 38% and 45% higher chance of not following EIBF compared to deliveries by vaginal institutions, respectively (Table 2). The chance of not being breastfed in deliveries at home was 8% lower compared to deliveries by vaginal institutions. The adjusted pooled prevalence ratio of EIBF in c-sections in public facilities was slightly higher in lower-middle income countries (0.650, 95% CI 0.635; 0.665) compared to low-income countries (0.544, 95% CI 0.521; 0.567) and upper-middle income countries (0.612 0.599; 0.626). Figure 1 illustrates the adjusted prevalence ratio by country and provides the heterogeneity coefficient according to the type of delivery and income groups. In general, aggregating the prevalence ratios shows that c-section deliveries greatly reduced the chances of not following EIBF compared to vaginal deliveries regardless of the income group.

**Table 2:** Crude and adjusted prevalence ratios of early initiation of breastfeeding stratified by type of delivery and place of birth according to income group for 73 countries from LMICs. Household health surveys^a^, 2010–2019.

Income group	Type and place of delivery	Prevalence ratio (PR)	
		Crude PR (95% CI)	Adj^b^ PR (95% CI)
All countries	Institutional vaginal delivery	Ref	Ref
(*n* = *73*)	Home vaginal delivery	0.927 (0.921; 0.934)	0.927 (0.920; 0.934)
	C-section public facilities	0.620 (0.610; 0.629)	0.618 (0.609; 0.628)
	C-section private facilities	0.550 (0.538; 0.563)	0.553 (0.541; 0.566)
Low-income	Institutional vaginal delivery	Ref	Ref
(*n* = *25*)	Home vaginal delivery	0.878 (0.868; 0.888)	0.879 (0.868; 0.890)
	C-section public facilities	0.542 (0.519; 0.565)	0.544 (0.521; 0.567)
	C-section private facilities	0.545 (0.497; 0.598)	0.552 (0.503; 0.605)
Lower-middle income	Institutional vaginal delivery	Ref	Ref
(*n* = *28*)	Home vaginal delivery	0.947 (0.937; 0.956)	0.954 (0.943; 0.964)
	C-section public facilities	0.658 (0.643; 0.673)	0.650 (0.635; 0.665)
	C-section private facilities	0.557 (0.542; 0.572)	0.555 (0.540; 0.571)
Upper-middle income	Institutional vaginal delivery	Ref	Ref
(*n* = *20*)	Home vaginal delivery	1.016 (0.995; 1.038)	0.987 (0.965; 1.010)
	C-section public facilities	0.609 (0.595; 0.622)	0.612 (0.599; 0.626)
	C-section private facilities	0.534 (0.511; 0.558)	0.547 (0.523; 0.572)

^a^Multiple Indicator Cluster Survey, Demographic Health Survey and National Health and Nutrition Survey (Ecuador).

^b^Adjusted for place of residence, maternal age, maternal formal education, wealth quintiles.

*C-section* cesarean section; *CI* confidence interval.

Though, most of the pooled adjusted estimates presented evidence of high heterogeneity across countries, with moderate heterogeneity for c-sections in private facilities between low-income countries (I^2^ = 69.9%) and home deliveries between countries of the upper middle income (I^2^ = 68.6%). In the meta-regression, a negative adjusted R^2^ was observed in most models, indicating that heterogeneity is affected by other variables but not the type of survey, with exception for home and public c-section deliveries for low-income countries, which the type of surveys explained 4.1% and 0.2% of the heterogeneity, respectively (Data not shown).

### National adjusted prevalence ratios of EIBF according to the type of delivery

In general, a decrease in the gradient of prevalence ratios of EIBF was observed according to type of delivery, being respectively higher for home deliveries, followed by public and private c-sections (Supplementary Table S3). This gradient was observed in 16 of 25 low-income countries, 15 of 28 lower-middle countries, and 11 of 20 upper-middle-income countries. The prevalence ratio of EIBF among children who born by c-section—regardless of whether they were in public or private facilities—reveal a higher chance of not having an EIBF compared to those children who born by institutional vaginal delivery. While some prevalence ratios of EIBF in children who were born by c-section in private facilities were not significant in some countries (8 out of 25 low-income; 3 out of 28 lower-middle income, and 3 out of 20 upper-middle income), in two upper-middle income countries (Suriname and Turkey) the probability of not having an EIBF in children who born by c-section compared to those who born by institutional vaginal delivery were significantly lower in public facilities than in private facilities. Surprisingly, in some lower-income countries (Bangladesh, Cameroon, and Pakistan) and upper-middle income countries (Gabon, and Jordan), the chances of having an EIBF in children who born at home were significantly higher compared to those who born by institutional vaginal deliveries.

## Discussion

The present study adds critical evidence of the inverse relationship between c-sections and EIBF, essentially in children born by c-section in private facilities in lower- and upper-middle income countries compared to institutional vaginal deliveries. This result was observed in a pooled analysis of 73 LMICs and remained significant at the national level for most countries (71%), even after adjustment for potential confounders. To our knowledge, this is the first multi-country analyses that investigated the association of EIBF and c-section by public and private health facilities.

Previous studies using multi-country data described similar findings of delay in EIBF among c-section births compared to institutional vaginal delivery^6,7,26^. In WHO Global Survey data of 373 health facilities and 244,569 singleton live births of low obstetric risk pregnancy, c-section was an independent factor associated with delay in EIBF^6^. In another study in 21 LMICs, although deliveries by c-section presented a significant delay in the median of EIBF, immediate skin-to-skin contact was associated with a shorter time for breastfeeding initiation in almost all countries^7^.

Evidence has shown that the inverse relationship between c-sections and EIBF indicates that optimal breastfeeding practices during the first years of life can be directly or indirectly affected by the mode of delivery. Analyses in LMICs countries showed that c-section deliveries increase the likelihood of feeding a child with prelacteal (feeding a child any liquid other than breast milk before breastfeeding is established)^9,27,28^. Many studies reported that children who were prelacteal fed are less likely to be exclusively breastfed under six months or to continue breastfeeding throughout the first year of life. Therefore, prelacteal feeding is a risk factor for suboptimal breastfeeding practices worldwide^29^. Importantly, the prevalence of EIBF and prelacteal feeding is inversely correlated in LMICs^30^.

Our results also showed that children born at home are also less likely to be breastfed in the first hour of birth compared to children who were born by vaginal delivery in a facility. In a pooled analysis of 298,656 women from 58 LMICs, a longer delay in EIBF was associated with c-section and home vaginal deliveries^26^. One possible explanation is that these infants receive prelacteal feeding (i.e., tea, water or infant formula) which have been reported to be associated with suboptimal breastfeeding practices, such as delayed in breastfeeding initiation^29^.

Even though the choice to breastfeed is subject to women’s choice, for many years this practice has declined around the world in detriment of the unconscionable and unregulated marketing of infant formula^31^. This gap in breastfeeding promotion changed healthcare attitudes and perception of how important it is for children to receive breast milk in the first hour after birth. C-section is a surgery procedure indicated in the circumstance of labor or high-risk pregnancy, but it should not be a barrier to promote early breastfeeding, and adequate support and trained personnel are essential to help women breastfeed after birth regardless of the type of delivery^32,33^. Most hospitals do not promote breastfeeding as part of health care. The adoption of the Baby Friendly Hospital Initiative as a facility-based intervention assures appropriate training to staff and medical providers to support mothers and their children’ success in breastfeeding^11,34^. Although public and private health facilities are encouraged to implement this initiative, more public facilities adhere to this initiative compared to the private sector^11^.

Our findings also reveal an inequity provision of c-sections, with a high prevalence of c-sections in richest countries, and a lower provision in low-income countries, either in public or private facilities. Previous analyses have shown that the global increase in c-section prevalence in the last three decades is markedly by within-country inequalities among LMICs^35–37^. In trend analysis from 2000 to 2015, c-section was almost five times more common in births in the richest versus the poorest quintiles in LMICs, higher in low-obstetric risk births among more educated women, and 1.6 times more frequent in private facilities than in public ones^10^. Furthermore, the marked difference in c-section prevalence between public and private facilities reveals unequal access to emergency obstetric care among the poorest subgroups and high levels of use of c-sections without medical indication in the richest subgroups, especially in middle and upper-middle income^10^.

Our analysis has important strengths, such as the inclusion of a large number of country data from LMICs with highly comparable nationally representative surveys in terms of definitions and methods, which enabled the investigation of four different categories of birth delivery and their association with EIBF. Datasets were built following a rigorous protocol to standardize those variables of birth and breastfeeding, and heterogeneity was not associated with the type of survey. In addition to descriptive ecological analysis, we conducted an analysis with individual data adjusting for individual characteristics. Besides, our study has some limitations. The major limitation is the fact that our dataset was composed of different years across the countries; however, we have selected only the most recent surveys from 2010 or later, which increases the reliability of our findings in the current scenario of increasing c-section rates in LMICs. We excluded countries with an insufficient number of observations in private c-sections and with an insufficient number of observations in private c-sections. High-income countries were not included in this study due to the small number of representative countries in our database and the absence of national surveys. We were also unable to explore whether institutional vaginal births occurred in private or public facilities.

To improve early initiation of breastfeeding prevalence at the national level it is essential to develop national breastfeeding policies based on the International Code of Marketing of Breast-milk Substitutes and institutionalization of the Baby-friendly Hospital Initiative^34,38^. Furthermore, antenatal breastfeeding counseling by peers or health personnel would increase timely and optimal breastfeeding practices in children born in health facilities. However, implementing health system interventions along with other interventions simultaneously combining other settings (i.e., home and community) may prevent children from being born at home, reducing the likelihood that they will receive options other than breastmilk^39^.

Since infants delivered by c-section are less likely to initiate breastfeeding within the first hour of life and the prevalence of c-section has increased globally, it is essential that the countries align with international recommendations that stipulate to keep the prevalence of c-section between 10 and 15% at the national level^40^. Health service personnel should be sensitized and adequately trained to reduce medically unnecessary use of c-sections, so as not to jeopardize the practice of breastfeeding, but also to avoid endangering, both the mother and her baby. Finally, women should be able to talk to health care providers and participate in decisions about their delivery, receiving adequate information, including risks and benefits^41^.

In conclusion, our findings indicate that in LMICs children born by c-section deliveries were less likely to receive EIBF compared to institutional vaginal deliveries, especially in private facilities compared to public facilities. A lower chance of EIBF was also observed in home vaginal deliveries compared to vaginal institutional deliveries. The relevance of EIBF must be promoted and adequate support must be provided to mothers successfully breastfed their children since the first hour after birth.

## Supplementary Information

Supplementary Information.

## Data Availability

The dataset supporting the conclusions of this article are publicity available at the respective surveys’ website described in the reference list.
