# Supplementary material for: Early initiation of breastfeeding is inversely associated with public and private c-sections in 73 lower- and middle-income countries
Source: Sci Rep. 2022 Dec 6;12:21081. doi: 10.1038/s41598-022-25564-w (PMC9727135; doi:10.1038/s41598-022-25564-w)
Supplement: Supplementary file 1 — Supplementary Information. [file 41598_2022_25564_MOESM1_ESM.docx]

SUPPLEMENTARY INFORMATION

**Early initiation of breastfeeding is inversely associated with public and private c-sections in 73 lower- and middle-income countries**

**Legends:**

**Supplementary Figure S1**. Flowchart of country data available for analysis

**Supplementary Table S1.** Weighed prevalence^1^ of type of delivery and place of birth for 73 countries from LMICs. Household health surveys, 2010-2019.

**Supplementary Table S2.** Weighed prevalence^1^ of early initiation of breastfeeding according to type of delivery and place of birth for 73 countries from LMICs. Household health surveys, 2010-2019.

**Supplementary Table S3**. Adjusted^1^ prevalence ratios (PR) of early initiation of breastfeeding by type of delivery and place of birth for 73 countries from LMICs. Household health surveys, 2010-2019.

**Supplementary Figure S1**. Flowchart of country data available for analysis

**Supplementary Table S1.** Weighed prevalence^1^ of type of delivery and place of birth for 73 countries from LMICs. Household health surveys, 2010-2019.

|  |  |  |  | **PREVALENCE OF TYPE OF DELIVERY AND PLACE OF BIRTH** | | | | | | | | | | | | |
| --- | --- | --- | --- | --- | --- | --- | --- | --- | --- | --- | --- | --- | --- | --- | --- | --- |
| **Country** | **Year** | **Source** | **N** | **Institutional vaginal delivery** | | |  | **Home vaginal delivery** | | |  | **Cesarean section public facilities** | |  | **Cesarean section private facilities** | |
|  |  |  |  | **%** | **95% CI** | |  | **%** | **95% CI** | |  | **%** | **95% CI** |  | **%** | **95% CI** |
| **LOW INCOME** |  |  |  |  |  |  |  |  |  |  |  |  |  |  |  |  |
| Afghanistan | 2015 | DHS | 12261 | 50.9 | (47.1;54.6) | |  | 45.5 | (41.9;49.2) | |  | 3.1 | (2.6;3.8) |  | 0.5 | (0.3;0.8) |
| Burundi | 2016 | DHS | 5470 | 79.8 | (78.2;81.3) | |  | 14.7 | (13.3;16.1) | |  | 5.0 | (4.3;5.9) |  | 0.5 | (0.4;0.8) |
| Benin | 2017 | DHS | 5733 | 79.9 | (77.9;81.8) | |  | 14.9 | (13.1;17.0) | |  | 4.0 | (3.4;4.6) |  | 1.2 | (0.9;1.5) |
| Burkina Faso | 2010 | DHS | 6024 | 71.1 | (68.2;73.8) | |  | 26.6 | (23.9;29.5) | |  | 2.0 | (1.6;2.5) |  | 0.2 | (0.1;0.6) |
| Central African Republic | 2010 | MICS | 4545 | 48.1 | (45.1;51.1) | |  | 47.5 | (44.4;50.6) | |  | 3.8 | (3;4.8.0) |  | 0.6 | (0.3;1.3) |
| Congo Democratic Republic | 2017 | MICS | 8559 | 78.4 | (76.0;80.6) | |  | 16.9 | (14.8;19.2) | |  | 3.1 | (2.4;4.1) |  | 1.6 | (1.0;2.4) |
| Comoros | 2012 | DHS | 1346 | 67.6 | (64.0;71.1) | |  | 21.5 | (18.3;25.1) | |  | 10.0 | (8.0;12.5) |  | 0.8 | (0.4;1.6) |
| Ethiopia | 2016 | DHS | 4242 | 33.7 | (29.8;37.7) | |  | 63.5 | (59.3;67.5) | |  | 2.5 | (2.0;3.3) |  | 0.3 | (0.2;0.5) |
| Gambia | 2018 | MICS | 3796 | 77.8 | (75.3;80.1) | |  | 18.5 | (16.3;20.9) | |  | 3.1 | (2.4;4.0) |  | 0.6 | (0.3;1.2) |
| Guinea-Bissau | 2018 | MICS | 2925 | 47.6 | (44.3;50.9) | |  | 49.5 | (46.1;53.0) | |  | 2.6 | (1.9;3.5) |  | 0.3 | (0.1;0.6) |
| Haiti | 2016 | DHS | 2541 | 30.6 | (27.7;33.5) | |  | 64.6 | (61.6;67.6) | |  | 3.4 | (2.7;4.4) |  | 1.4 | (0.9;2.0) |
| Cambodia | 2014 | DHS | 2970 | 80.4 | (77.9;82.6) | |  | 11.4 | (9.4;13.7) | |  | 4.7 | (3.8;5.9) |  | 3.5 | (2.7;4.6) |
| Liberia | 2013 | DHS | 3195 | 56.3 | (52.7;59.8) | |  | 38.9 | (35.1;42.9) | |  | 3.9 | (3.0;5.1) |  | 0.9 | (0.5;1.7) |
| Madagascar | 2018 | MICS | 5237 | 36.7 | (34.2;39.4) | |  | 61.3 | (58.6;63.9) | |  | 1.3 | (1.0;1.7) |  | 0.7 | (0.4;1.1) |
| Mali | 2018 | DHS | 4089 | 67.3 | (63.6;70.8) | |  | 30.2 | (26.7;34.0) | |  | 2.2 | (1.8;2.9) |  | 0.2 | (0.1;0.4) |
| Mozambique | 2011 | DHS | 4771 | 53.2 | (50.4;55.9) | |  | 42.8 | (40.0;45.7) | |  | 3.8 | (3.2;4.5) |  | 0.2 | (0.1;0.5) |
| Malawi | 2015 | DHS | 6879 | 85.9 | (84.6;87.1) | |  | 7.2 | (6.3;8.3) | |  | 5.7 | (5.0;6.5) |  | 1.2 | (0.8;1.7) |
| Niger | 2012 | DHS | 4945 | 32.6 | (29.9;35.4) | |  | 65.9 | (63.0;68.6) | |  | 1.5 | (1.1;2.0) |  | 0.1 | (0.0;0.3) |
| Nepal | 2016 | DHS | 1957 | 52.7 | (49.5;55.8) | |  | 37.1 | (33.8;40.6) | |  | 6.1 | (4.9;7.6) |  | 4.1 | (3.0;5.6) |
| Rwanda | 2014 | DHS | 3274 | 77.8 | (76.1;79.4) | |  | 8.3 | (7.1;9.7) | |  | 13.3 | (12.0;14.8) |  | 0.5 | (0.3;0.9) |
| Sierra Leone | 2017 | MICS | 4744 | 73.3 | (71.0;75.4) | |  | 23.4 | (21.4;25.6) | |  | 2.8 | (2.2;3.4) |  | 0.5 | (0.3;0.9) |
| Chad | 2014 | DHS | 6806 | 22.8 | (20.9;24.8) | |  | 75.6 | (73.5;77.6) | |  | 1.5 | (1.1;2.1) |  | 0.1 | (0.0;0.2) |
| Togo | 2017 | MICS | 1988 | 71.3 | (67.7;74.7) | |  | 20.0 | (16.7;23.8) | |  | 7.4 | (5.8;9.3) |  | 1.3 | (0.8;2.1) |
|  |  |  |  |  |  | |  |  |  | |  |  |  |  |  |  |
| *Cont.* |  |  |  |  | | | | | | | | | | | | |
|  |  |  |  | **PREVALENCE OF TYPE OF DELIVERY AND PLACE OF BIRTH** | | | | | | | | | | | | |
| **Country** | **Year** | **Source** | **N** | **Institutional vaginal delivery** | | |  | **Home vaginal delivery** | | |  | **Cesarean section public facilities** | |  | **Cesarean section private facilities** | |
|  |  |  |  | **%** | **95% CI** | |  | **%** | **95% CI** | |  | **%** | **95% CI** |  | **%** | **95% CI** |
| Tanzania | 2015 | DHS | 4397 | 58.0 | (55.2;60.8) | |  | 35.2 | (32.2;38.3) | |  | 4.4 | (3.7;5.3) |  | 2.4 | (1.8;3.1) |
| Uganda | 2016 | DHS | 6297 | 69.2 | (67.3;71.1) | |  | 23.5 | (21.6;25.4) | |  | 5.0 | (4.3;5.8) |  | 2.3 | (1.8;3.0) |
| **LOWER-MIDDLE INCOME** |  |  |  |  |  | |  |  |  | |  |  |  |  |  |  |
| Bangladesh | 2019 | MICS | 9285 | 17.4 | (16.5;18.4) | |  | 46.6 | (45.3;47.9) | |  | 5.6 | (5.1;6.2) |  | 30.4 | (29.2;31.6) |
| Cote d’Ivoire | 2016 | MICS | 3749 | 66.5 | (63.6;69.3) | |  | 30.2 | (27.4;33.2) | |  | 2.4 | (1.9;3.2) |  | 0.9 | (0.5;1.5) |
| Cameroon | 2018 | DHS | 3978 | 63.6 | (60.6;66.5) | |  | 32.1 | (29.1;35.3) | |  | 2.7 | (2.2;3.4) |  | 1.6 | (1.2;2.1) |
| Egypt | 2014 | DHS | 6745 | 31.0 | (29.5;32.6) | |  | 11.4 | (10.2;12.7) | |  | 12.5 | (11.4;13.7) |  | 45.1 | (43.4;46.8) |
| Ghana | 2017 | MICS | 3466 | 65.0 | (62.4;67.5) | |  | 22.1 | (19.6;24.8) | |  | 10.7 | (9.3;12.2) |  | 2.2 | (1.6;3.0) |
| Guatemala | 2014 | DHS | 4940 | 38.6 | (36.7;40.6) | |  | 32.1 | (29.6;34.7) | |  | 18.8 | (17.3;20.5) |  | 10.4 | (9.3;11.7) |
| Guyana | 2014 | MICS | 1258 | 75.7 | (72.4;78.8) | |  | 7.3 | (5.4;9.8) | |  | 11.1 | (9.0;13.6) |  | 5.9 | (4.5;7.7) |
| Honduras | 2011 | DHS | 4570 | 66.3 | (64.4;68.2) | |  | 14.9 | (13.6;16.4) | |  | 16.5 | (15.1;18.1) |  | 2.2 | (1.7;2.8) |
| Indonesia | 2017 | DHS | 7083 | 64.0 | (62.2;65.7) | |  | 16.9 | (15.4;18.4) | |  | 7.2 | (6.5;8.0) |  | 12.0 | (10.9;13.1) |
| India | 2015 | DHS | 1E+05 | 63.0 | (62.5;63.5) | |  | 17.9 | (17.5;18.3) | |  | 7.2 | (6.9;7.4) |  | 11.9 | (11.5;12.3) |
| Kenya | 2014 | DHS | 8220 | 56.3 | (54.6;58.1) | |  | 34.7 | (33.0;36.4) | |  | 5.9 | (5.1;6.8) |  | 3.1 | (2.6;3.8) |
| Lao PDR | 2017 | MICS | 4460 | 58.6 | (56.2;61.0) | |  | 35.5 | (32.9;38.1) | |  | 5.1 | (4.4;6.0) |  | 0.8 | (0.5;1.2) |
| Lesotho | 2018 | MICS | 1175 | 73.2 | (69.6;76.5) | |  | 9.1 | (7.3;11.3) | |  | 11.8 | (9.4;14.7) |  | 5.9 | (4.3;8.1) |
| Myanmar | 2015 | DHS | 1918 | 24.5 | (21.6;27.8) | |  | 54.6 | (50.7;58.4) | |  | 16.1 | (14.0;18.5) |  | 4.8 | (3.5;6.4) |
| Mongolia | 2018 | MICS | 2199 | 72.2 | (69.4;74.9) | |  | 1.6 | (0.9;2.8) | |  | 25.0 | (22.4;27.9) |  | 1.1 | (0.6;2.1) |
| Mauritania | 2015 | MICS | 4172 | 64.4 | (61.4;67.3) | |  | 30.7 | (27.7;33.8) | |  | 4.5 | (3.7;5.5) |  | 0.4 | (0.2;0.8) |
| Nigeria | 2018 | DHS | 13302 | 38.0 | (36.3;39.7) | |  | 59.0 | (57.3;60.8) | |  | 1.3 | (1.0;1.5) |  | 1.7 | (1.4;2.1) |
| Pakistan | 2017 | DHS | 4149 | 45.6 | (43.0;48.3) | |  | 28.5 | (25.4;31.9) | |  | 6.3 | (5.1;7.7) |  | 19.5 | (17.2;22.1) |
| Philippines | 2017 | DHS | 4043 | 67.6 | (64.9;70.2) | |  | 16.7 | (14.7;19.0) | |  | 8.6 | (7.4;10.0) |  | 7.1 | (5.3;9.3) |
| Papua New Guinea | 2016 | DHS | 3744 | 52.6 | (48.8;56.4) | |  | 43.8 | (39.8;47.9) | |  | 3.6 | (2.4;5.3) |  | 0.1 | (0.0;0.2) |
| State of Palestine | 2014 | MICS | 2891 | 79.0 | (77.4;80.5) | |  | 0.7 | (0.4;1.1) | |  | 12.3 | (11.1;13.6) |  | 8.0 | (7.0;9.1) |
|  |  |  |  |  |  | |  |  |  | |  |  |  |  |  |  |
|  |  |  |  |  | | | | | | | | | | | | |
| *Cont.* |  |  |  |  | | | | | | | | | | | | |
|  |  |  |  | **PREVALENCE OF TYPE OF DELIVERY AND PLACE OF BIRTH** | | | | | | | | | | | | |
| **Country** | **Year** | **Source** | **N** | **Institutional vaginal delivery** | | |  | **Home vaginal delivery** | | |  | **Cesarean section public facilities** | |  | **Cesarean section private facilities** | |
|  |  |  |  | **%** | **95% CI** | |  | **%** | **95% CI** | |  | **%** | **95% CI** |  |  | **95% CI** |
| Sudan | 2014 | MICS | 5684 | 18.6 | (16.7;20.7) | |  | 72.3 | (69.8;74.6) | |  | 8.4 | (7.3;9.5) |  | 0.7 | (0.4;1.1) |
| El Salvador | 2014 | MICS | 2832 | 66.0 | (63.6;68.4) | |  | 2.5 | (1.8;3.3) | |  | 29.9 | (27.6;32.3) |  | 1.6 | (1.1;2.3) |
| Eswatini | 2014 | MICS | 987 | 76.1 | (72.8;79.1) | |  | 12.3 | (10.1;14.8) | |  | 7.4 | (5.9;9.3) |  | 4.2 | (2.8;6.3) |
| Tunisia | 2018 | MICS | 1212 | 56.1 | (52.9;59.2) | |  | 0.3 | (0.1;0.8) | |  | 28.2 | (25.5;31.1) |  | 15.4 | (13.0;18.1) |
| Yemen | 2013 | DHS | 6527 | 26.3 | (24.5;28.1) | |  | 67.9 | (65.8;69.9) | |  | 3.2 | (2.7;3.8) |  | 2.6 | (2.1;3.3) |
| Zambia | 2018 | DHS | 4083 | 80.0 | (78.0;81.9) | |  | 13.7 | (11.9;15.7) | |  | 5.3 | (4.2;6.7) |  | 0.9 | (0.6;1.5) |
| Zimbabwe | 2019 | MICS | 2337 | 76.7 | (74.4;78.9) | |  | 14.5 | (12.5;16.8) | |  | 6.4 | (5.4;7.6) |  | 2.4 | (1.8;3.3) |
| **UPPER-MIDDLE INCOME** |  |  |  |  |  | |  |  |  | |  |  |  |  |  |  |
| Angola | 2015 | DHS | 6150 | 42.8 | (40.4;45.2) | |  | 53.2 | (50.7;55.6) | |  | 3.9 | (3.2;4.8) |  | 0.1 | (0.0;0.4) |
| Albania | 2017 | DHS | 1083 | 67.3 | (63.4;70.9) | |  | 1.1 | (0.5;2.1) | |  | 29.2 | (25.9;32.7) |  | 2.5 | (1.2;5.0) |
| Belize | 2015 | MICS | 916 | 61.9 | (57.5;66.1) | |  | 3.6 | (2.3;5.6) | |  | 30.2 | (26.2;34.5) |  | 4.3 | (2.8;6.5) |
| Colombia | 2010 | DHS | 6672 | 57.7 | (56.1;59.2) | |  | 4.6 | (4;5.4.0) | |  | 37.6 | (36.1;39.2) |  | 0.1 | (0.0;0.3) |
| Costa Rica | 2018 | MICS | 1286 | 69.7 | (64.8;74.1) | |  | 2.2 | (1.2;4.0) | |  | 24.3 | (20.3;28.9) |  | 3.8 | (2.1;6.8) |
| Dominican Republic | 2014 | MICS | 7566 | 39.7 | (38.0;41.4) | |  | 2.1 | (1.7;2.7) | |  | 32.9 | (31.3;34.5) |  | 25.3 | (23.7;26.9) |
| Ecuador | 2012 | NSS | 4141 | 51.4 | (49.0;53.8) | |  | 7.9 | (6.6;9.3) | |  | 25.2 | (23.1;27.3) |  | 15.6 | (13.6;17.9) |
| Gabon | 2012 | DHS | 2649 | 80.8 | (78.1;83.3) | |  | 8.4 | (7.0;10.1) | |  | 5.6 | (4.2;7.5) |  | 5.1 | (3.6;7.4) |
| Iraq | 2018 | MICS | 6250 | 53.4 | (50.6;56.2) | |  | 13.4 | (11.9;15.1) | |  | 22.4 | (20.7;24.1) |  | 10.8 | (9.3;12.5) |
| Jordan | 2017 | DHS | 4293 | 70.6 | (68.2;73.0) | |  | 1.1 | (0.7;1.8) | |  | 17.2 | (15.6;19.0) |  | 11.1 | (9.2;13.2) |
| Maldives | 2016 | DHS | 1193 | 52.5 | (48.9;56.0) | |  | 4.9 | (3.5;6.9) | |  | 32.1 | (28.6;35.8) |  | 10.5 | (8.2;13.5) |
| Mexico | 2015 | MICS | 3032 | 56.1 | (52.6;59.5) | |  | 3.1 | (2.1;4.4) | |  | 29.5 | (26.8;32.4) |  | 11.3 | (9.0;14.1) |
| Namibia | 2013 | DHS | 2121 | 72.4 | (70.1;74.6) | |  | 12.0 | (10.4;13.8) | |  | 11.9 | (10.5;13.5) |  | 3.7 | (2.6;5.4) |
| Panama | 2013 | MICS | 2278 | 63.5 | (58.9;67.9) | |  | 8.8 | (7.0;10.9) | |  | 22.7 | (19.5;26.3) |  | 5.0 | (2.9;8.6) |
| Peru | 2018 | DHS | 8487 | 56.6 | (55.1;58.2) | |  | 7.5 | (6.6;8.5) | |  | 25.8 | (24.5;27.1) |  | 10.1 | (9.1;11.2) |
|  |  |  |  |  |  | |  |  |  | |  |  |  |  |  |  |
|  |  |  |  |  |  | |  |  |  | |  |  |  |  |  |  |
|  |  |  |  |  |  | |  |  |  | |  |  |  |  |  |  |
| *Cont.* |  |  |  |  |  | |  |  |  | |  |  |  |  |  |  |
|  |  |  |  | **PREVALENCE OF TYPE OF DELIVERY AND PLACE OF BIRTH** | | | | | | | | | | | | |
| **Country** | **Year** | **Source** | **N** | **Institutional vaginal delivery** | | |  | **Home vaginal delivery** | | |  | **Cesarean section public facilities** | |  | **Cesarean section private facilities** | |
|  |  |  |  | **%** | **95% CI** | |  | **%** | **95% CI** | |  | **%** | **95% CI** |  |  | **95% CI** |
| Paraguay | 2016 | MICS | 1803 | 47.3 | (44.0;50.7) | |  | 6.8 | (5.2;8.8) | |  | 32.9 | (30.1;35.9) |  | 13.0 | (10.8;15.5) |
| Suriname | 2018 | MICS | 1393 | 76.0 | (73.1;78.7) | |  | 6.8 | (5.2;8.9) | |  | 9.9 | (8.1;12.1) |  | 7.2 | (5.5;9.4) |
| Thailand | 2019 | MICS | 3915 | 64.4 | (60.4;68.2) | |  | 1.0 | (0.6;1.6) | |  | 28.5 | (24.9;32.3) |  | 6.1 | (4.6;8.1) |
| Turkey | 2013 | DHS | 1469 | 51.5 | (48.0;54.9) | |  | 1.6 | (1.1;2.5) | |  | 22.2 | (19.7;25.1) |  | 24.6 | (21.6;28.0) |
| South Africa | 2016 | DHS | 1406 | 70.8 | (67.3;74.0) | |  | 3.9 | (2.8;5.4) | |  | 20.6 | (17.8;23.7) |  | 4.7 | (3.3;6.7) |

^1^ Weighed for national population of women of reproductive age (15-49 years); Source: United Nations, 2019

CI = confidence interval; MICS = Multiple Indicator Cluster Survey; DHS = Demographic Health Survey; NSS = National Health and Nutrition Survey.

**Supplementary Table S2.** Weighed prevalence^1^ of early initiation of breastfeeding according to type of delivery and place of birth for 73 countries from LMICs. Household health surveys, 2010-2019.

|  |  |  |  |  | **PREVALENCE OF EARLY INITIATION OF BREASTFEEDING** | | | | | | | | | | | |
| --- | --- | --- | --- | --- | --- | --- | --- | --- | --- | --- | --- | --- | --- | --- | --- | --- |
| **Country** | **Year** | **Source** | **All** | | **Institutional vaginal delivery** | | | **Home vaginal delivery** | | | **Cesarean section public facilities** | | | **Cesarean section private facilities** | | |
|  |  |  | **%** | **95% CI** |  | **95% CI** | | **%** | **95% CI** | | **%** | **95% CI** | | **%** | **95% CI** | |
| **LOW INCOME** |  |  |  |  |  |  |  |  |  |  |  |  |  |  |  |  |
| Afghanistan | 2015 | DHS | 40.9 | (38.5;43.4) | 43.2 | (40.8;45.7) | | 39.8 | (36.1;43.6) | | 24.5 | (16.2;35.3) | | 15.6 | (4.0;44.9) | |
| Burundi | 2016 | DHS | 85.0 | (83.8;86.2) | 89.3 | (88.1;90.4) | | 80.2 | (76.4;83.4) | | 36.2 | (29.8;43.2) | | 26.8 | (11.0;52.1) | |
| Benin | 2017 | DHS | 54.1 | (52.1;56.0) | 55.1 | (52.9;57.3) | | 58.4 | (54.0;62.6) | | 23.9 | (17.8;31.4) | | 28.3 | (18.0;41.6) | |
| Burkina Faso | 2010 | DHS | 42.1 | (40.1;44.2) | 44.7 | (42.3;47.2) | | 35.6 | (32.4;38.9) | | 36.2 | (28.1;45.3) | | 49.5 | (9.5;90.2) | |
| Central African Republic | 2010 | MICS | 43.5 | (41.0;46.0) | 45.1 | (41.9;48.3) | | 43.4 | (40.1;46.7) | | 29.0 | (21.9;37.3) | | 21.8 | (7.2;50.0) | |
| Congo Democratic Republic | 2017 | MICS | 46.9 | (43.8;50.1) | 50.0 | (46.3;53.7) | | 41.7 | (36.7;46.8) | | 15.0 | (8.4;25.4) | | 14.1 | (5.2;33.0) | |
| Comoros | 2012 | DHS | 33.7 | (29.8;37.9) | 36.7 | (32.5;41.2) | | 32.7 | (24.7;41.9) | | 18.2 | (10.1;30.4) | | 10.2 | (0.8;61.5) | |
| Ethiopia | 2016 | DHS | 73.3 | (71.0;75.6) | 76.7 | (73.1;80.0) | | 73.1 | (70.1;75.9) | | 37.0 | (23.8;52.5) | | 40.2 | (19.1;65.7) | |
| Gambia | 2018 | MICS | 46.5 | (44.2;48.7) | 49.3 | (46.6;52.0) | | 41.9 | (37.6;46.3) | | 9.4 | (3.8;21.6) | | 11.1 | (0.8;64.7) | |
| Guinea Bissau | 2018 | MICS | 46.3 | (44.0;48.7) | 49.6 | (46.1;53.0) | | 44.7 | (41.7;47.8) | | 21.0 | (11.5;35.3) | | 23.2 | (0.1;98.8) | |
| Haiti | 2016 | DHS | 47.0 | (44.4;49.6) | 49.3 | (44.9;53.7) | | 48.1 | (44.9;51.4) | | 20.9 | (13.1;31.8) | | 6.3 | (1.6;21.6) | |
| Cambodia | 2014 | DHS | 62.6 | (60.1;65.0) | 68.1 | (65.6;70.5) | | 49.0 | (41.4;56.7) | | 31.9 | (23.4;41.9) | | 18.3 | (10.6;29.6) | |
| Liberia | 2013 | DHS | 61.2 | (57.4;65.0) | 63.8 | (59.4;68.0) | | 61.6 | (55.8;67.1) | | 22.5 | (13.6;34.8) | | 38.8 | (11.4;75.8) | |
| Madagascar | 2018 | MICS | 45.2 | (43.1;47.3) | 49.1 | (45.9;52.4) | | 44.0 | (41.5;46.5) | | 12.1 | (5.2;25.8) | | 5.8 | (1.0;27.1) | |
| Mali | 2018 | DHS | 63.7 | (61.3;66.0) | 68.0 | (65.3;70.7) | | 57.1 | (53.2;61.0) | | 22.2 | (13.5;34.4) | | 44.1 | (12.7;81.0) | |
| Mozambique | 2011 | DHS | 76.7 | (74.8;78.4) | 78.0 | (75.7;80.2) | | 76.4 | (73.6;79.0) | | 59.4 | (50.2;67.9) | | 91.6 | (81.4;96.5) | |
| Malawi | 2015 | DHS | 76.3 | (74.9;77.6) | 78.9 | (77.5;80.3) | | 67.8 | (62.1;73.0) | | 52.8 | (46.6;58.8) | | 43.2 | (28.8;58.8) | |
| Niger | 2012 | DHS | 52.9 | (50.3;55.6) | 69.9 | (66.3;73.3) | | 44.9 | (41.7;48.1) | | 36.1 | (23.6;50.9) | | 45.6 | (0.0;100.0) | |
| Nepal | 2016 | DHS | 55.0 | (51.9;57.9) | 67.0 | (63.4;70.4) | | 47.0 | (41.6;52.5) | | 26.1 | (18.1;36.1) | | 16.0 | (7.5;30.9) | |
| Rwanda | 2014 | DHS | 80.5 | (79.0;81.9) | 87.9 | (86.4;89.2) | | 71.0 | (64.9;76.4) | | 42.7 | (38.0;47.4) | | 61.3 | (33.3;83.4) | |
|  |  |  |  |  |  |  | |  |  | |  |  | |  |  | |
| *Cont.* |  |  |  |  |  |  | |  |  | |  |  | |  |  | |
|  |  |  | **PREVALENCE OF EARLY INITIATION OF BREASTFEEDING** | | | | | | | | | | | | | |
| **Country** | **Year** | **Source** | **All** | | **Institutional vaginal delivery** | | | **Home vaginal delivery** | | | **Cesarean section public facilities** | | | **Cesarean section private facilities** | | |
|  |  |  | **%** | **95% CI** |  | **95% CI** | | **%** | **95% CI** | | **%** | **95% CI** | | **%** | **95% CI** | |
| Sierra Leone | 2017 | MICS | 55.7 | (53.4;57.9) | 57.8 | (55.3;60.2) | | 53.8 | (49.8;57.8) | | 23.1 | (15.7;32.5) | | 22.1 | (6.9;52.1) | |
| Chad | 2014 | DHS | 23.0 | (20.8;25.4) | 22.9 | (19.6;26.6) | | 23.4 | (20.9;26.0) | | 6.5 | (2.3;16.7) | | 18.4 | (0.0;100.0) | |
| Togo | 2017 | MICS | 48.3 | (45.5;51.2) | 53.3 | (49.9;56.6) | | 44.0 | (38.2;49.9) | | 15.1 | (8.9;24.6) | | 32.3 | (16.0;54.4) | |
| Tanzania | 2015 | DHS | 51.2 | (48.8;53.5) | 62.0 | (59.4;64.5) | | 40.0 | (36.2;43.9) | | 11.5 | (6.9;18.5) | | 21.5 | (12.5;34.5) | |
| Uganda | 2016 | DHS | 66.1 | (64.4;67.7) | 71.2 | (69.3;73.1) | | 59.9 | (56.8;62.9) | | 35.1 | (28.9;41.7) | | 40.7 | (30.0;52.3) | |
| **LOWER-MIDDLE INCOME** |  |  |  |  |  |  | |  |  | |  |  | |  |  | |
| Bangladesh | 2019 | MICS | 46.6 | (45.4;47.7) | 50.9 | (48.1;53.7) | | 63.0 | (61.4;64.5) | | 24.2 | (20.2;28.6) | | 23.1 | (21.5;24.8) | |
| Cote d`Ivoire | 2016 | MICS | 36.6 | (33.8;39.5) | 36.5 | (33.2;39.9) | | 39.2 | (34.7;43.8) | | 17.1 | (8.5;31.6) | | 12.8 | (3.2;39.0) | |
| Cameroon | 2018 | DHS | 48.4 | (46.0;50.8) | 50.1 | (47.2;53.0) | | 47.6 | (43.8;51.4) | | 24.6 | (16.0;35.8) | | 35.8 | (24.3;49.3) | |
| Egypt | 2014 | DHS | 27.1 | (25.6;28.7) | 36.8 | (34.1;39.5) | | 47.7 | (43.4;52.0) | | 18.1 | (15.0;21.6) | | 17.6 | (15.7;19.7) | |
| Ghana | 2017 | MICS | 52.0 | (49.4;54.6) | 58.8 | (55.5;62.0) | | 46.5 | (42.0;51.1) | | 25.1 | (19.4;31.7) | | 35.8 | (20.6;54.5) | |
| Guatemala | 2014 | DHS | 63.1 | (61.2;65.0) | 72.5 | (69.8;75.1) | | 81.3 | (78.3;84.0) | | 34.5 | (30.9;38.4) | | 25.0 | (20.5;30.3) | |
| Guyana | 2014 | MICS | 49.2 | (45.4;53.0) | 55.8 | (51.6;59.9) | | 42.3 | (32.7;52.6) | | 26.8 | (17.7;38.3) | | 15.2 | (8.5;25.6) | |
| Honduras | 2011 | DHS | 63.8 | (61.9;65.7) | 74.5 | (72.3;76.5) | | 81.3 | (77.7;84.3) | | 12.1 | (9.2;15.7) | | 7.3 | (3.0;16.5) | |
| Indonesia | 2017 | DHS | 56.7 | (55.0;58.3) | 62.7 | (60.8;64.6) | | 56.3 | (52.8;59.6) | | 36.4 | (31.4;41.7) | | 37.1 | (32.7;41.8) | |
| India | 2015 | DHS | 41.5 | (41.0;42.1) | 45.9 | (45.3;46.5) | | 34.6 | (33.5;35.7) | | 38.4 | (36.4;40.4) | | 30.5 | (29.1;32.0) | |
| Kenya | 2014 | DHS | 62.2 | (60.0;64.4) | 66.3 | (63.3;69.2) | | 62.4 | (58.8;66.0) | | 35.9 | (28.2;44.4) | | 28.0 | (15.2;45.7) | |
| Lao | 2017 | MICS | 50.1 | (47.8;52.3) | 55.3 | (52.6;58.0) | | 45.9 | (42.3;49.6) | | 25.3 | (19.4;32.3) | | 3.7 | (0.8;15.7) | |
| Lesotho | 2018 | MICS | 56.4 | (52.8;60.1) | 63.3 | (59.5;67.1) | | 55.4 | (43.2;67.1) | | 22.7 | (16.1;31.0) | | 39.8 | (24.3;57.7) | |
| Myanmar | 2015 | DHS | 66.8 | (63.6;69.8) | 73.5 | (67.9;78.5) | | 68.2 | (64.1;72.0) | | 52.5 | (44.8;60.0) | | 64.6 | (49.7;77.1) | |
| Mongolia | 2018 | MICS | 70.2 | (67.2;73.0) | 80.1 | (77.1;82.8) | | 65.1 | (33.0;87.6) | | 43.1 | (36.8;49.6) | | 45.7 | (13.3;82.3) | |
| Mauritania | 2015 | MICS | 61.8 | (59.0;64.6) | 62.2 | (58.7;65.5) | | 67.2 | (63.4;70.8) | | 22.0 | (15.2;30.7) | | 45.8 | (13.4;82.1) | |
| Nigeria | 2018 | DHS | 42.1 | (40.8;43.5) | 52.3 | (50.4;54.2) | | 36.7 | (35.1;38.4) | | 23.1 | (17.2;30.3) | | 18.1 | (12.9;25.0) | |
|  |  |  |  |  |  |  | |  |  | |  |  | |  |  | |
| *Cont.* |  |  |  |  |  |  | |  |  | |  |  | |  |  | |
|  |  |  | **PREVALENCE OF EARLY INITIATION OF BREASTFEEDING** | | | | | | | | | | | | | |
| **Country** | **Year** | **Source** | **All** | | **Institutional vaginal delivery** | | | **Home vaginal delivery** | | | **Cesarean section public facilities** | | | **Cesarean section private facilities** | | |
|  |  |  | **%** | **95% CI** |  | **95% CI** | | **%** | **95% CI** | | **%** | **95% CI** | | **%** | **95% CI** | |
| Pakistan | 2017 | DHS | 19.6 | (17.3;22.0) | 23.1 | (20.0;26.4) | | 23.8 | (19.6;28.6) | | 10.8 | (6.7;16.8) | | 7.9 | (5.5;11.3) | |
| Philippines | 2017 | DHS | 56.9 | (54.3;59.4) | 61.9 | (58.9;64.8) | | 55.0 | (49.6;60.4) | | 37.8 | (30.4;45.8) | | 37.3 | (23.0;54.4) | |
| Papua New Guinea | 2016 | DHS | 53.1 | (50.3;55.9) | 53.0 | (49.6;56.3) | | 56.0 | (51.9;59.9) | | 20.2 | (9.5;38.0) | | 58.4 | (0.2;99.9) | |
| State of Palestine | 2014 | MICS | 40.8 | (38.5;43.2) | 48.5 | (45.9;51.1) | | 27.2 | (9.5;57.0) | | 9.3 | (6.6;13.0) | | 15.2 | (11.3;20.2) | |
| Sudan | 2014 | MICS | 68.7 | (66.7;70.7) | 73.3 | (69.8;76.5) | | 70.7 | (68.4;73.0) | | 43.9 | (38.1;49.9) | | 36.7 | (14.3;67.0) | |
| El Salvador | 2014 | MICS | 42.0 | (39.5;44.6) | 53.1 | (50.0;56.3) | | 66.7 | (53.8;77.5) | | 16.8 | (14.1;19.9) | | 15.7 | (6.1;34.8) | |
| Eswatini | 2014 | MICS | 48.3 | (44.3;52.4) | 53.7 | (48.9;58.5) | | 39.4 | (30.0;49.6) | | 24.2 | (15.4;35.8) | | 19.7 | (8.3;39.9) | |
| Tunisia | 2018 | MICS | 31.6 | (28.5;34.8) | 38.7 | (34.5;43.1) | | 40.2 | (0.8;98.3) | | 19.7 | (15.3;25.0) | | 27.1 | (20.3;35.3) | |
| Yemen | 2013 | DHS | 52.9 | (50.8;55.0) | 50.6 | (47.5;53.8) | | 56.4 | (54.1;58.8) | | 24.5 | (18.1;32.2) | | 18.1 | (10.8;28.6) | |
| Zambia | 2018 | DHS | 75.5 | (73.4;77.5) | 81.3 | (79.3;83.1) | | 64.7 | (59.3;69.8) | | 22.1 | (15.1;31.1) | | 25.4 | (13.7;42.1) | |
| Zimbabwe | 2019 | MICS | 59.3 | (57.0;61.6) | 67.8 | (65.4;70.2) | | 39.5 | (33.5;45.9) | | 18.1 | (12.1;26.1) | | 15.8 | (7.9;29.2) | |
| **UPPER-MIDDLE INCOME** |  |  |  |  |  |  | |  |  | |  |  | |  |  | |
| Angola | 2015 | DHS | 48.3 | (45.7;50.9) | 52.7 | (49.2;56.3) | | 46.4 | (43.2;49.5) | | 26.2 | (17.3;37.5) | | 21.8 | (0.0;100.0) | |
| Albania | 2017 | DHS | 56.5 | (51.8;61.1) | 63.7 | (57.8;69.3) | | 80.4 | (0.1;100.0) | | 38.7 | (32.8;45) | | 62.9 | (19.4;92.3) | |
| Belize | 2015 | MICS | 68.3 | (64.3;72.0) | 75.3 | (70.6;79.4) | | 75.0 | (59.5;85.9) | | 54.7 | (46.3;62.8) | | 57.0 | (41.1;71.6) | |
| Colombia | 2010 | DHS | 63.4 | (61.9;64.9) | 74.0 | (72.2;75.8) | | 69.3 | (63.4;74.5) | | 46.5 | (44.0;49.0) | | 8.0 | (0.0;100.0) | |
| Costa Rica | 2018 | MICS | 52.9 | (48.2;57.6) | 65.9 | (60.1;71.3) | | 63.1 | (29.8;87.3) | | 18.2 | (12.0;26.6) | | 31.6 | (10.1;65.7) | |
| Dominican Republic | 2014 | MICS | 38.1 | (36.1;40.1) | 53.4 | (50.5;56.3) | | 34.8 | (25.2;45.9) | | 33.5 | (30.6;36.6) | | 20.2 | (17.5;23.2) | |
| Ecuador | 2012 | NSS | 52.6 | (49.8;55.4) | 61.5 | (57.5;65.4) | | 71.5 | (64.8;77.4) | | 40.4 | (35.7;45.2) | | 33.2 | (27.7;39.3) | |
| Gabon | 2012 | DHS | 32.3 | (28.5;36.5) | 34.3 | (29.8;39.2) | | 40.3 | (32.3;49.0) | | 13.4 | (5.5;29.1) | | 7.9 | (2.1;25.8) | |
| Iraq | 2018 | MICS | 32.4 | (29.9;34.9) | 44.2 | (41.0;47.4) | | 46.7 | (39.4;54.0) | | 8.3 | (6.4;10.7) | | 5.9 | (3.9;8.8) | |
| Jordan | 2017 | DHS | 67.0 | (64.4;69.6) | 74.3 | (71.5;77.0) | | 86.1 | (49.6;97.5) | | 45.0 | (39.6;50.5) | | 51.8 | (43.2;60.2) | |
| Maldives | 2016 | DHS | 66.5 | (62.3;70.4) | 68.9 | (63.3;74.0) | | 48.4 | (32.3;64.8) | | 66.6 | (60.2;72.5) | | 62.1 | (46.5;75.6) | |
|  |  |  |  |  |  |  | |  |  | |  |  | |  |  | |
|  |  |  |  |  |  |  | |  |  | |  |  | |  |  | |
| *Cont.* |  |  |  |  |  |  | |  |  | |  |  | |  |  | |
|  |  |  | **PREVALENCE OF EARLY INITIATION OF BREASTFEEDING** | | | | | | | | | | | | | |
| **Country** | **Year** | **Source** | **All** | | **Institutional vaginal delivery** | | | **Home vaginal delivery** | | | **Cesarean section public facilities** | | | **Cesarean section private facilities** | | |
|  |  |  | **%** | **95% CI** |  | **95% CI** | | **%** | **95% CI** | | **%** | **95% CI** | | **%** | **95% CI** | |
| Mexico | 2015 | MICS | 51.0 | (47.6;54.5) | 61.2 | (57.0;65.2) | | 66.2 | (50.3;79.2) | | 39.9 | (35.2;44.8) | | 25.7 | (15.0;40.3) | |
| Namibia | 2013 | DHS | 71.2 | (68.9;73.4) | 74.6 | (71.9;77.0) | | 75.6 | (69.6;80.8) | | 52.4 | (44.8;59.9) | | 52.4 | (34.8;69.5) | |
| Panama | 2013 | MICS | 47.0 | (43.3;50.7) | 53.8 | (49.2;58.2) | | 71.0 | (63.8;77.2) | | 25.0 | (18.7;32.6) | | 18.5 | (5.5;47.0) | |
| Peru | 2018 | DHS | 50.5 | (49.1;52.0) | 72.2 | (70.5;73.9) | | 74.8 | (69.8;79.1) | | 11.3 | (9.7;13.0) | | 9.4 | (6.6;13.1) | |
| Paraguay | 2016 | MICS | 49.5 | (46.2;52.9) | 61.6 | (56.7;66.3) | | 54.6 | (43.6;65.3) | | 39.0 | (33.5;44.7) | | 29.7 | (21.8;39.0) | |
| Suriname | 2018 | MICS | 52.1 | (48.2;55.8) | 58.2 | (53.8;62.5) | | 51.1 | (39.0;63.1) | | 20.9 | (13.7;30.4) | | 30.9 | (19.8;44.7) | |
| Thailand | 2019 | MICS | 34.0 | (30.3;37.9) | 41.0 | (36.2;46.1) | | 7.8 | (2.9;19.4) | | 21.9 | (17.2;27.5) | | 20.4 | (9.9;37.4) | |
| Turkey | 2013 | DHS | 64.5 | (61.3;67.5) | 69.9 | (65.3;74.1) | | 55.9 | (0.6;99.6) | | 53.0 | (46.1;59.8) | | 63.9 | (57.2;70.1) | |
| South Africa | 2016 | DHS | 67.3 | (64.0;70.4) | 70.5 | (66.7;74.0) | | 61.7 | (42.8;77.6) | | 60.9 | (54.2;67.3) | | 51.6 | (38.0;65.0) | |

^1^ Weighed for national population of women of reproductive age (15-49 years); Source: World Bank, 2020

CI = confidence interval; MICS = Multiple Indicator Cluster Survey; DHS = Demographic Health Survey; NSS = National Health and Nutrition Survey.

**Supplementary Table S3**. Adjusted^1^ prevalence ratios (PR) of early initiation of breastfeeding by type of delivery and place of birth for 73 countries from LMICs. Household health surveys, 2010-2019.

| **Country** | **Year** | **Source** | **Institutional vaginal delivery** | **Home vaginal delivery** | | | **Cesarean section public facilities** | | | **Cesarean section private facilities** | | |
| --- | --- | --- | --- | --- | --- | --- | --- | --- | --- | --- | --- | --- |
|  |  |  |  | **Adj PR** | **95% CI** | | **Adj PR** | **95% CI** | | **Adj PR** | **95% CI** | |
| **LOW INCOME** |  |  |  |  |  | |  |  | |  |  | |
| Afghanistan | 2015 | DHS | Ref. | 1.009 | (0.969;1.052) | | 0.581 | (0.476;0.709) | | 0.300 | (0.122;0.739) | |
| Burundi | 2016 | DHS | Ref. | 0.894 | (0.862;0.928) | | 0.410 | (0.353;0.475) | | 0.346 | (0.206;0.582) | |
| Benin | 2017 | DHS | Ref. | 1.012 | (0.945;1.083) | | 0.420 | (0.325;0.541) | | 0.504 | (0.335;0.757) | |
| Burkina Faso | 2010 | DHS | Ref. | 0.757 | (0.698;0.821) | | 0.664 | (0.504;0.874) | | 0.842 | (0.347;2.041) | |
| Central African Republic | 2010 | MICS | Ref. | 0.890 | (0.824;0.962) | | 0.711 | (0.561;0.900) | | 0.453 | (0.208;0.984) | |
| Congo Democratic Republic | 2017 | MICS | Ref. | 0.817 | (0.772;0.865) | | 0.447 | (0.336;0.595) | | 0.364 | (0.211;0.626) | |
| Comoros | 2012 | DHS | Ref. | 0.869 | (0.711;1.061) | | 0.428 | (0.284;0.644) | | 0.265 | (0.043;1.630) | |
| Ethiopia | 2016 | DHS | Ref. | 0.928 | (0.886;0.971) | | 0.561 | (0.453;0.695) | | 0.460 | (0.308;0.686) | |
| Gambia | 2018 | MICS | Ref. | 0.929 | (0.848;1.017) | | 0.238 | (0.128;0.440) | | 0.197 | (0.031;1.266) | |
| Guinea Bissau | 2018 | MICS | Ref. | 0.829 | (0.758;0.908) | | 0.378 | (0.230;0.622) | | 0.317 | (0.052;1.949) | |
| Haiti | 2016 | DHS | Ref. | 0.933 | (0.847;1.028) | | 0.436 | (0.288;0.661) | | 0.225 | (0.077;0.656) | |
| Cambodia | 2014 | DHS | Ref. | 0.725 | (0.645;0.814) | | 0.453 | (0.349;0.587) | | 0.394 | (0.28;0.555) | |
| Liberia | 2013 | DHS | Ref. | 0.926 | (0.869;0.986) | | 0.506 | (0.372;0.688) | | 0.500 | (0.258;0.967) | |
| Madagascar | 2018 | MICS | Ref. | 0.858 | (0.802;0.919) | | 0.262 | (0.131;0.524) | | 0.154 | (0.041;0.581) | |
| Mali | 2018 | DHS | Ref. | 0.844 | (0.796;0.895) | | 0.327 | (0.224;0.478) | | 0.713 | (0.386;1.315) | |
| Mozambique | 2011 | DHS | Ref. | 0.938 | (0.903;0.974) | | 0.783 | (0.696;0.880) | | 1.112 | (0.897;1.378) | |
| Malawi | 2015 | DHS | Ref. | 0.836 | (0.783;0.893) | | 0.696 | (0.637;0.761) | | 0.608 | (0.484;0.764) | |
| Niger | 2012 | DHS | Ref. | 0.709 | (0.668;0.752) | | 0.482 | (0.360;0.645) | | 0.691 | (0.199;2.403) | |
| Nepal | 2016 | DHS | Ref. | 0.720 | (0.658;0.787) | | 0.399 | (0.292;0.545) | | 0.290 | (0.178;0.473) | |
| Rwanda | 2014 | DHS | Ref. | 0.801 | (0.738;0.869) | | 0.483 | (0.432;0.539) | | 0.593 | (0.393;0.893) | |
| Sierra Leone | 2017 | MICS | Ref. | 0.945 | (0.890;1.003) | | 0.388 | (0.274;0.549) | | 0.318 | (0.115;0.882) | |
| Chad | 2014 | DHS | Ref. | 1.088 | (0.962;1.231) | | 0.258 | (0.109;0.608) | | 0.749 | (0.119;4.700) | |
|  |  |  |  |  |  | |  |  | |  |  | |
| *Cont.* |  |  |  |  |  | |  |  | |  |  | |
| **Country** | **Year** | **Source** | **Institutional vaginal delivery** | **Home vaginal delivery** | | | **Cesarean section public facilities** | | | **Cesarean section private facilities** | | |
|  |  |  |  | **Adj PR** | **95% CI** | | **Adj PR** | **95% CI** | | **95% CI** | **Adj PR** | |
| Togo | 2017 | MICS | Ref. | 0.800 | (0.713;0.898) | | 0.290 | (0.191;0.439) | | 0.592 | (0.330;1.062) | |
| Tanzania | 2015 | DHS | Ref. | 0.708 | (0.659;0.761) | | 0.194 | (0.130;0.292) | | 0.298 | (0.188;0.472) | |
| Uganda | 2016 | DHS | Ref. | 0.855 | (0.815;0.897) | | 0.470 | (0.399;0.553) | | 0.495 | (0.390;0.630) | |
| **LOWER-MIDDLE INCOME** |  |  |  |  |  | |  |  | |  |  | |
| Bangladesh | 2019 | MICS | Ref. | 1.203 | (1.140;1.269) | | 0.461 | (0.391;0.545) | | 0.456 | (0.420;0.495) | |
| Cote d`Ivoire | 2016 | MICS | Ref. | 1.015 | (0.924;1.114) | | 0.559 | (0.359;0.871) | | 0.692 | (0.292;1.643) | |
| Cameroon | 2018 | DHS | Ref. | 0.966 | (0.886;1.052) | | 0.390 | (0.266;0.572) | | 0.692 | (0.493;0.971) | |
| Egypt | 2014 | DHS | Ref. | 1.243 | (1.129;1.369) | | 0.469 | (0.402;0.547) | | 0.444 | (0.402;0.490) | |
| Ghana | 2017 | MICS | Ref. | 0.764 | (0.702;0.831) | | 0.458 | (0.386;0.543) | | 0.613 | (0.439;0.856) | |
| Guatemala | 2014 | DHS | Ref. | 1.057 | (1.014;1.102) | | 0.489 | (0.446;0.536) | | 0.358 | (0.303;0.422) | |
| Guyana | 2014 | MICS | Ref. | 0.782 | (0.640;0.955) | | 0.427 | (0.315;0.577) | | 0.342 | (0.202;0.580) | |
| Honduras | 2011 | DHS | Ref. | 1.025 | (0.983;1.068) | | 0.149 | (0.120;0.185) | | 0.108 | (0.053;0.220) | |
| Indonesia | 2017 | DHS | Ref. | 0.934 | (0.884;0.986) | | 0.527 | (0.468;0.594) | | 0.547 | (0.491;0.609) | |
| India | 2015 | DHS | Ref. | 0.897 | (0.879;0.914) | | 0.833 | (0.807;0.859) | | 0.621 | (0.600;0.643) | |
| Kenya | 2014 | DHS | Ref. | 0.934 | (0.885;0.986) | | 0.574 | (0.480;0.686) | | 0.391 | (0.262;0.583) | |
| Lao | 2017 | MICS | Ref. | 0.837 | (0.782;0.895) | | 0.515 | (0.415;0.638) | | 0.143 | (0.038;0.543) | |
| Lesotho | 2018 | MICS | Ref. | 0.856 | (0.708;1.035) | | 0.410 | (0.305;0.551) | | 0.508 | (0.348;0.742) | |
| Myanmar | 2015 | DHS | Ref. | 0.989 | (0.917;1.068) | | 0.697 | (0.612;0.793) | | 0.716 | (0.567;0.904) | |
| Mongolia | 2018 | MICS | Ref. | 0.706 | (0.501;0.994) | | 0.573 | (0.520;0.630) | | 0.533 | (0.307;0.925) | |
| Mauritania | 2015 | MICS | Ref. | 1.026 | (0.966;1.090) | | 0.285 | (0.208;0.390) | | 0.692 | (0.381;1.256) | |
| Nigeria | 2018 | DHS | Ref. | 0.876 | (0.836;0.918) | | 0.366 | (0.271;0.495) | | 0.357 | (0.266;0.480) | |
| Pakistan | 2017 | DHS | Ref. | 1.180 | (1.065;1.306) | | 0.481 | (0.364;0.636) | | 0.353 | (0.279;0.447) | |
| Philippines | 2017 | DHS | Ref. | 0.880 | (0.821;0.944) | | 0.649 | (0.568;0.742) | | 0.637 | (0.523;0.776) | |
| Papua New Guinea | 2016 | DHS | Ref. | 1.027 | (0.955;1.104) | | 0.441 | (0.314;0.619) | | 1.293 | (0.567;2.946) | |
| State of Palestine | 2014 | MICS | Ref. | 0.557 | (0.264;1.177) | | 0.197 | (0.144;0.270) | | 0.301 | (0.222;0.408) | |
|  |  |  |  |  |  | |  |  | |  |  | |
|  |  |  |  |  |  | |  |  | |  |  | |
| *Cont.* |  |  |  |  |  | |  |  | |  |  | |
| **Country** | **Year** | **Source** | **Institutional vaginal delivery** | **Home vaginal delivery** | | | **Cesarean section public facilities** | | | **Cesarean section private facilities** | | |
|  |  |  |  | **Adj PR** | **95% CI** | | **Adj PR** | **95% CI** | | **Adj PR** | **95% CI** | |
| Sudan | 2014 | MICS | Ref. | 1.041 | (0.991;1.094) | | 0.547 | (0.485;0.617) | | 0.507 | (0.325;0.792) | |
| El Salvador | 2014 | MICS | Ref. | 1.070 | (0.895;1.278) | | 0.360 | (0.311;0.418) | | 0.354 | (0.194;0.647) | |
| Eswatini | 2014 | MICS | Ref. | 0.777 | (0.629;0.959) | | 0.426 | (0.286;0.633) | | 0.366 | (0.189;0.710) | |
| Tunisia | 2018 | MICS | Ref. | 0.929 | (0.321;2.685) | | 0.471 | (0.371;0.599) | | 0.667 | (0.503;0.885) | |
| Yemen | 2013 | DHS | Ref. | 1.154 | (1.093;1.219) | | 0.566 | (0.457;0.702) | | 0.336 | (0.233;0.482) | |
| Zambia | 2018 | DHS | Ref. | 0.765 | (0.716;0.818) | | 0.299 | (0.232;0.385) | | 0.355 | (0.205;0.617) | |
| Zimbabwe | 2019 | MICS | Ref. | 0.557 | (0.485;0.639) | | 0.293 | (0.214;0.401) | | 0.245 | (0.135;0.445) | |
| **UPPER-MIDDLE INCOME** |  |  |  |  |  |  |  |  |  |  |  |  |
| Angola | 2015 | DHS | Ref. | 0.902 | (0.841;0.967) | | 0.469 | (0.355;0.618) | | 0.748 | (0.254;2.204) | |
| Albania | 2017 | DHS | Ref. | 1.044 | (0.702;1.552) | | 0.528 | (0.445;0.627) | | 0.688 | (0.384;1.232) | |
| Belize | 2015 | MICS | Ref. | 0.864 | (0.667;1.119) | | 0.688 | (0.610;0.775) | | 0.696 | (0.511;0.949) | |
| Colombia | 2010 | DHS | Ref. | 1.008 | (0.956;1.062) | | 0.645 | (0.616;0.677) | | 0.546 | (0.189;1.573) | |
| Costa Rica | 2018 | MICS | Ref. | 0.882 | (0.652;1.195) | | 0.227 | (0.177;0.293) | | 0.231 | (0.103;0.519) | |
| Dominican Republic | 2014 | MICS | Ref. | 0.719 | (0.602;0.860) | | 0.624 | (0.585;0.666) | | 0.431 | (0.390;0.477) | |
| Ecuador | 2012 | NSS | Ref. | 1.044 | (0.974;1.119) | | 0.697 | (0.647;0.750) | | 0.551 | (0.484;0.629) | |
| Gabon | 2012 | DHS | Ref. | 1.231 | (1.072;1.413) | | 0.539 | (0.369;0.789) | | 0.344 | (0.163;0.726) | |
| Iraq | 2018 | MICS | Ref. | 1.025 | (0.942;1.115) | | 0.197 | (0.166;0.234) | | 0.155 | (0.113;0.212) | |
| Jordan | 2017 | DHS | Ref. | 1.162 | (1.009;1.338) | | 0.613 | (0.565;0.665) | | 0.607 | (0.535;0.689) | |
| Maldives | 2016 | DHS | Ref. | 0.900 | (0.734;1.104) | | 0.886 | (0.816;0.962) | | 0.874 | (0.731;1.046) | |
| Mexico | 2015 | MICS | Ref. | 1.000 | (0.867;1.154) | | 0.648 | (0.593;0.708) | | 0.436 | (0.356;0.533) | |
| Namibia | 2013 | DHS | Ref. | 1.018 | (0.941;1.102) | | 0.766 | (0.685;0.857) | | 0.768 | (0.590;0.998) | |
| Panama | 2013 | MICS | Ref. | 1.080 | (0.984;1.186) | | 0.425 | (0.351;0.513) | | 0.391 | (0.237;0.647) | |
| Peru | 2018 | DHS | Ref. | 0.948 | (0.901;0.997) | | 0.177 | (0.158;0.198) | | 0.159 | (0.123;0.205) | |
|  |  |  |  |  |  | |  |  | |  |  | |
|  |  |  |  |  |  | |  |  | |  |  | |
| **Country** | **Year** | **Source** | **Institutional vaginal delivery** | **Home vaginal delivery** | | | **Cesarean section public facilities** | | | **Cesarean section private facilities** | | |
|  |  |  |  | **Adj PR** | **95% CI** | | **Adj PR** | **95% CI** | | **Adj PR** | **95% CI** | |
| Paraguay | 2016 | MICS | Ref. | 0.935 | (0.807;1.083) | | 0.679 | (0.609;0.758) | | 0.483 | (0.395;0.591) | |
| Suriname | 2018 | MICS | Ref. | 0.918 | (0.764;1.102) | | 0.428 | (0.325;0.563) | | 0.583 | (0.439;0.776) | |
| Thailand | 2019 | MICS | Ref. | 0.294 | (0.148;0.584) | | 0.594 | (0.533;0.663) | | 0.359 | (0.246;0.524) | |
| Turkey | 2013 | DHS | Ref. | 0.856 | (0.618;1.186) | | 0.683 | (0.604;0.773) | | 0.838 | (0.752;0.933) | |
| South Africa | 2016 | DHS | Ref. | 0.921 | (0.745;1.138) | | 0.803 | (0.718;0.898) | | 0.680 | (0.512;0.901) | |

^1^ Adjusted for living area, maternal education, maternal age, wealth quintiles.

CS = cesarean section; MICS = Multiple Indicator Cluster Survey; DHS = Demographic Health Survey; NSS = National Health and Nutrition Survey.
